# SMMDB: a web-accessible database for small molecule modulators and their targets involved in neurological diseases

**DOI:** 10.1093/database/bay082

**Published:** 2018-09-13

**Authors:** Subodh Kumar Mishra, Neha Jain, Uma Shankar, Arpita Tawani, Amit Mishra, Amit Kumar

**Affiliations:** 1 Discipline of Biosciences and Biomedical Engineering, Indian Institute of Technology Indore, Indore, Simrol, India; 2 Cellular and Molecular Neurobiology Unit, Indian Institute of Technology Jodhpur, Rajasthan, India

## Abstract

High-throughput screening and better understanding of small molecule’s structure–activity relationship (SAR) using computational biology techniques have greatly expanded the face of drug discovery process in better discovery of therapeutics for various disease. Small Molecule Modulators Database (SMMDB) includes >1100 small molecules that have been either approved by US Food and Drug Administration, are under investigation or were rejected in clinical trial for any kind of neurological diseases. The comprehensive information about small molecules includes the details about their molecular targets (such as protein or enzyme, DNA, RNA, antisense RNA etc.), pharmacokinetic and pharmacodynamic properties such as binding affinity to their targets (K_d_, K_i_, IC_50_ and EC_50_ if available), mode of action, log *P*-value, number of hydrogen bond donor and acceptors, their clinical trial status, their 2D and three-dimensional structures etc. To enrich the basic annotation of every small molecule entry present in SMMDB, it is hyperlinked to their description present in PubChem, DrugBank, PubMed and KEGG database. The annotation about their molecular targets was enriched by linking it with UniProt and GenBank and STRING database that can be utilized to study the interaction and relation between various targets involved in single neurological disease. All molecules present in the SMMDB are made available to download in single file and can be further used in establishing the SAR, structure-based drug designing as well as shape-based virtual screening for developing the novel therapeutics against neurological diseases. The scope of this database majorly covers the interest of scientific community and researchers who are engaged in putting their endeavor toward therapeutic development and investigating the pathogenic mechanism of various neurological diseases. The graphical user interface of the SMMDB is accessible on http://bsbe.iiti.ac.in/bsbe/smmdb.

## Introduction

Neuron, the structural and functional unit of human nervous system, forms a complex circuit in the body that transmits different signals to perform the cellular activities. Any defect in their function leads to various structural or biochemical abnormalities in the brain, spinal cord, and neurons that cause neurological disorders (1–4). Majority of neurological diseases are characterized by some of the following processes like protein aggregation, inclusion body formation, defect in cellular stress management, abnormality in DNA repair mechanism, expansion of certain set of nucleotide repeat in genes, deregulation in RNA processing and microRNA (mi-RNA) regulatory pathway, etc. (5–13).

Modern medicine and advanced combinatorial chemistry owe much of its progress toward disease treatments and cures by developing small molecules as therapeutic candidates. Owing to their low molecular weight (<1000 Da), they get easily dissolve and absorbed in gastrointestinal tract following its oral administration. Most of the orally active compounds that have achieved Phase II clinical trial status follow the Lipinski’s rule of five (i.e., molecular weight ≤ 500 Daltons; log *P* ≤ 5; H-bond donors ≤5; H-bond acceptors ≤10) (14,15) with few exceptions of flavonoids, alkaloids, and antibiotics. Interestingly, small molecules with the polar surface area of ≤60–70 Å act within the central nervous system very efficiently (16). Moreover, if the sum of nitrogen and oxygen atoms is ≤5, their probability to cross the blood–brain barrier increases significantly (17). Small molecules having >10 rotatable bonds are suspected for the poor bioavailability (18). Such molecules can penetrate the blood–brain barrier and provide a leap forward in therapeutic developments for the psychological and neurological disorders. Additionally, drugs that discriminately act at neuronal versus peripheral sites typically produce fewer treatment-related adverse events.

Small molecules have been evolved as a potential tool todevelop novel and effective therapeutics for the past several decades. Current research efforts to estimate the potential of a small molecule as promising therapeutic agents largely deal with the molecular targets that are involved in disease pathology like DNA, transcripts, epigenetic regulators, proteins and ion channels (19–23). For example, several small molecules with different targets have been employed as therapeutic for Alzheimer’s disease (AD). Verubecestat (MK-8931) is a potent inhibitor of beta-secretase amyloid precursor protein cleaving enzyme that is required for the production of β-amyloid (Aβ) peptides (24). Bryostatin has been shown to restore the synaptic loss in the animal model of AD by activating the protein kinase C (PKC) epsilon (PKC*ɛ*) (25). Up-regulation of PKC decreases the production of Aβ as well as hyperphosphorylation of tau proteins. Other molecules act as an inhibitor of Phosphodiesterases such as Rolipram (26) and Sildenafil (27). Methylthioninium chloride is shown to reduce the aggregation of tau protein in-vitro as well as *in vivo* (28). Similarly, in case of amyotrophic lateral sclerosis (ALS), there are several small molecules that are being under investigation to test their therapeutic potential by targeting many class of molecular target.

Typically, the drug discovery process involved the considerable components of trials, positive hits and errors that lead to lots of information not only about a successful drug but also for those molecules that failed to become a drug or promising therapeutics. Further this information play important role while investigating the same macromolecule as drug target by developing an improved pharmacophore model for optimal molecular recognition. Recently, because of the development of high throughput screening and advancement in the molecular biology techniques, literature about such small molecules that have been studied as therapeutics for the neurological disorders are publishing rapidly in scientific community in electronic formats (29–33). However, such published information in electronic journals imposes several restrictions such as unavailability of data in the ready-to-use format for numerical analysis and lack of structural query platform (34). A drug-discovery process can be accelerated by providing these missing features and filling the gaps between these information resources in literature and pharmaceutical research.

As yet, few of the databases have been available to expedite researchers to gather information about drugs that target various diseases such as Therapeutic Target Database having a data set of therapeutic proteins and nucleic acid targets (35). However, it does not focus on many neuropathological conditions like neurofibromatosis type 2, Charcot–Marie–Tooth disease, Niemann–Pick disease, Kleine–Levin syndrome and Spinocerebellar Ataxia. Moreover, it also missed the updated information about small molecules and their drug target in various neurological diseases. Such as in case ALS to date, >50 small molecules have been studied for their therapeutic potential by targeting >30 kinds of molecular targets (see the supplementary Table 1 for the complete list of small molecule and their targets). Small molecule modulators of RNA (SMMRNA) is another database that only contains the information about such small molecules that selectively binds and regulates the RNA (36). DrugBank is another knowledgebase that possesses detailed information about small molecules/drugs, their targets and mechanism of action. However, it did not contain updated information about many neurological conditions. For example, it lacks the complete information of those molecules that have been studied as therapeutics for curing the ALS disease. On searching the DrugBank for ALS disease, it gave a total of 88 small molecules/drug hits and 5 drug targets hits. However, out of 88 drugs hits only 12 small molecule hits were found to be a true positive. Comparative Toxicogenomics Database (CTD) is a database that contains information about the effect of environmental chemicals/small molecules on the human health. However, on searching the therapeutic molecules for ALS disease in CTD database, it only gave 20 hits that seem to be incomplete (see the supplementary Table 1). Kyoto Encyclopedia of Genes and Genomes (KEGG) disease database is also lacking the updated information about therapeutic molecules for various neurological diseases. For example, there are only two small molecules are available in KEGG disease database as a therapeutics for the ALS disease (entry ID: H00058) and no drugs are available as therapeutics for Fragile X syndrome (FXS, entry ID: H00465).

Therefore, to the best our knowledge currently no database is available that specifically focuses on such small molecules that able to modulate the activity of several classes of molecular targets and thereby has potential to affect the neurological diseased condition. These molecular targets can be enzymes, receptors, transcriptional regulatory proteins, m-RNA, etc. To conclude this comprehensive set of data on a single console, we have constructed Small Molecule Modulators Database (SMMDB).

SMMDB is a unique portal that provides inclusive information about small molecules that have been explored as potent therapeutics for neurological disorders. Specifically, SMMDB provides detailed pharmacodynamics and pharmacokinetic properties of these small molecules such as experimentally determined binding data (K_d_, K_i_, IC_50_ and EC_50_) of small molecule with their targets, their 2D and three-dimensional (3D) structures, and their molecular descriptor’s and drug likeness properties such as molecular formula, molecular mass, AlogP, drug generic name, canonical simplified molecular-input line-entry (SMILE) and smiles arbitrary target specification (SMARTS) notation, formal charge, molecular solubility and polar surface, dipole magnitude, average bond length, number of rotatable bonds, number of hydrogen bond donor and acceptor, number of chains, number of aromatic rings, radius of gyration, their PubChem, DrugBank, KEGG Drug database identifiers etc.

The graphical user interface (GUI) of SMMDB is built on very simple and portable indexing that makes it accessible to browse on several platforms. Our database also provides an efficient searching method that includes full-text searching, advanced search options as well as structure-based search tool. SMMDB allows the downloading of 2D and 3D structure and energetically minimized 3D conformation (min 255 conformations for every molecule) of all small molecules present in the database. Using the application programming interface (API) of STRING database, SMMDB also provides the interactome of molecular targets available in the database.

This large conformation library of small molecule and annotated information about their targets would be useful in establishing SAR and shape-based virtual screening for development of novel therapeutics against neurological diseases. We believe that comprehensive information about such small molecules on a single platform would benefit the scientific community in understanding the pathogenic mechanism as well as the development of effective therapeutic approaches for various neurological disorders.

## Materials and methods

### Data compendium and manual curation

The comprehensive information about SMMDB’s small molecules supported by the experimental evidence present in the openly accessible peer-reviewed journals that were available on PubMed and Google Scholar. The manual curation process involved the searching of various keywords on the PubMed and google scholar, such as ‘neurological disease name and small molecule’, ‘neurological disorder and drugs’, ‘approved drugs for the neurological disease’ and ‘small molecules under investigation for particular neurological disorders’ to get the related literature. The full text (including abstract, material and method, introduction, result and discussion section) of all the obtained literature was reviewed to filter out the false positive search results. The example of keyword searching in PubMed was demonstrated in Supplementary Table S2. Once the screens were verified, the comprehensive information about small molecules such as details of small molecules, their molecular targets and binding interactions such as [association constant (K_a_) and dissociation constant (K_d_)]) were extracted from the full text of original scientific literature. The PubMed ID of each respective article was hyperlinked in the browsing list of database. The DrugBank, PubChem compound and KEGG Drug ID of small molecule and UniProt, GenBank and UniGene ID of targets were gathered from the respective database and hyperlinked to the original database. For each molecular target, PDB database was mined and linked with its PDB ID where-so-ever available. These provided links would be helpful in consulting the more information about small molecules and their targets.

### Database overview

The database was constructed using XAMPP (version 5.6.20) open source package (under the GPL license), which simultaneously supports MySQL, Apache, and PHP and provides an integrated database development platform. Apache2 was used as a web server platform and MySQL-RDMS (relational database management system) (5.6.24-MySQL Community Server) was used as the database server for data storing; organization and query execution (see Supplementary Tables S3 and S4 for a complete description of The MySQL database). SMMDB web server is running on the Dell Inc. (Model # PowerEdge R720xd) system, which is equipped with Intel(R) Xeon(R) E5–26650@2.40 GHz processor. Website pages were built using PHP language on Net Beans IDE (8.1) platform. The tables in the database were showed using DataTables plug-in by executing the jQueries written in AJAX code in the respective PHP pages. The DataTables plugin have several advantages such as easy and attractive pagination, instant search engine, multicolumn ordering and wide variety of extension. The SMMDB site is best viewed by Google Chrome, Firefox and Opera browser enabled with minimum version of Java.

### Structure and molecular properties of ligands

Structures of ligands were drawn using and Discovery studio 4.0 and saved as sdf file format. The structure of each ligand and their abbreviated R groups were built using Discovery Studio 4.0 (Accelerys Inc., San Diego, CA) and Marvin Sketch and save in sdf format. Discovery studio 4.0 was used to fix the bad valence if any and clean the 3D geometry of each small molecule structure imported in the database. The molecular descriptors for each ligand were calculated in Discovery Studio 4.0. The molecular formula of each ligand was displayed in PubChem format while molecular weight and strain energy were displayed in g/mol and kcal/mol units, respectively.

### JSME installation and structure search tool

Structure editor tool was built using Java applet based Successor of Java Molecular Editor (JSME) platform. This program facilitates the input of query molecule either by interactive drawing options or uploading the query structure from local disc space. To initialize the JSME in the webpage, initializing functions and placeholder were written within the JavaScript. The JSME distributions were downloaded and install in a local disc that contains a freely available bootstrap JavaScript file and highly optimized JavaScript code to detect the browser type, select the matching HTML file and load it.

### Advanced search options

Advance search form provides the eight types of molecular properties (molecular mass, AlogP, molecular weight, number of hydrogen bond donor and acceptor, number of aromatic bonds, molecular polar surface area, and number of rotatable bonds) as a search option to filter the database molecule. The user can select either a single search parameter or all eight parameters at a time as search criteria using ‘And’ or ‘OR’ operator and filter the database molecule according to their choice.

### 3D conformer generation

The high-quality 3D conformers of each ligand/drug in the database were created in Discovery Studio 4.0 using ‘generate conformation’ protocol. The ‘Best’ conformation method was chosen for the generation of 3D conformers of ligand that delivers better conformational coverage among all the available methods and conformers generated by this method could be further used for the 3D pharmacophore generation. These conformers were minimized using a SMART minimizer method by Merck Molecular Force Field (MMFF) with an energy threshold of 20 kcal/mol. A maximum of 255 conformations were generated for each molecule with RMS gradient of 0.1 (kcal/mol per Å).

### Embedding the STRING API

We used the API available at STRING database to show and search the interacting protein partner of a particular drug targets available in the SMMDB (37). The required code was written in the python script and accessed through the HTTP request.

**Figure 1:**
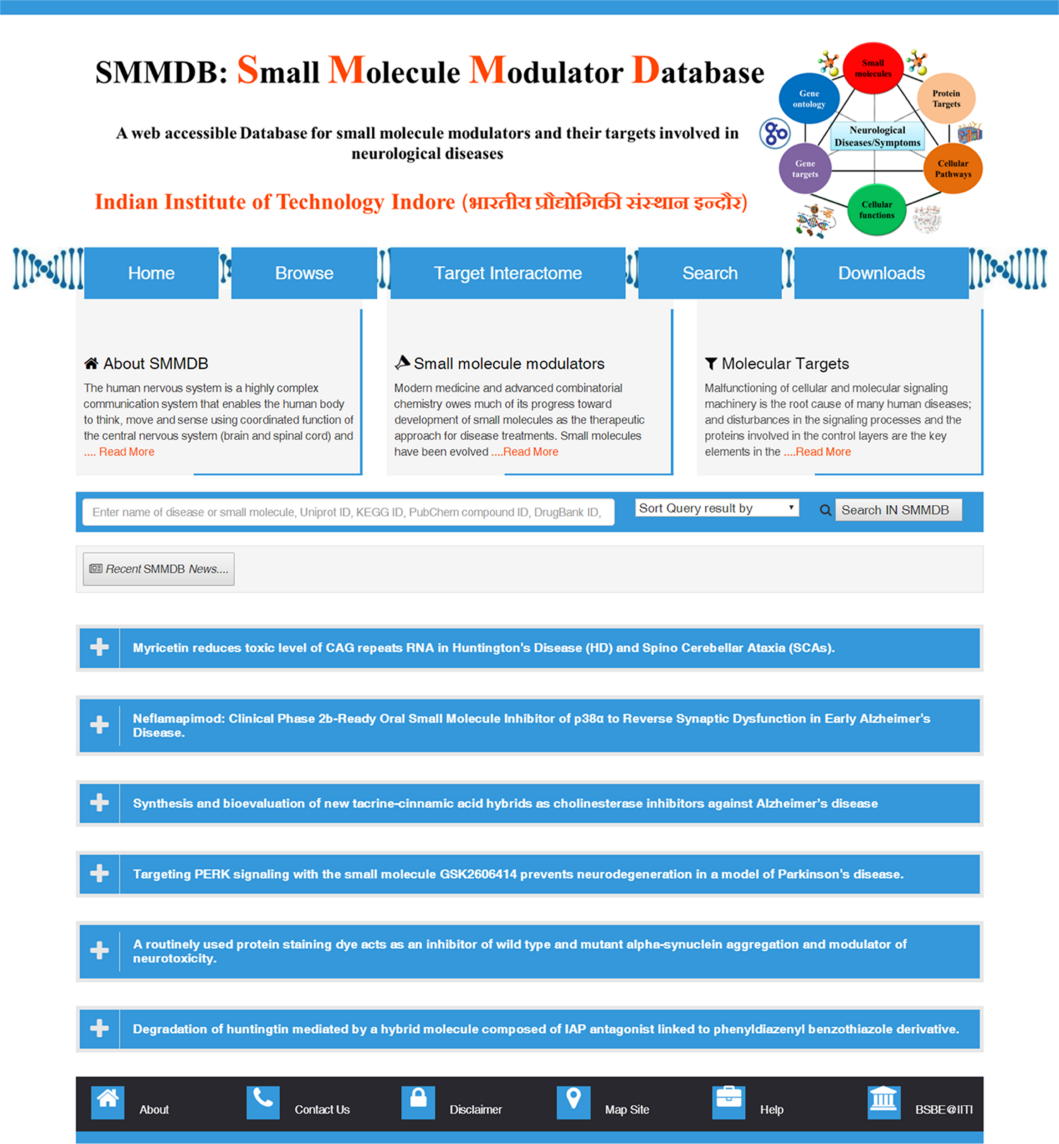
Home page of SMMDB (those involved as therapeutics in neurological disease). Screenshot Illustrating the browse, search and download options.

## Results

### Database architecture and data mining

**Figure 2:**
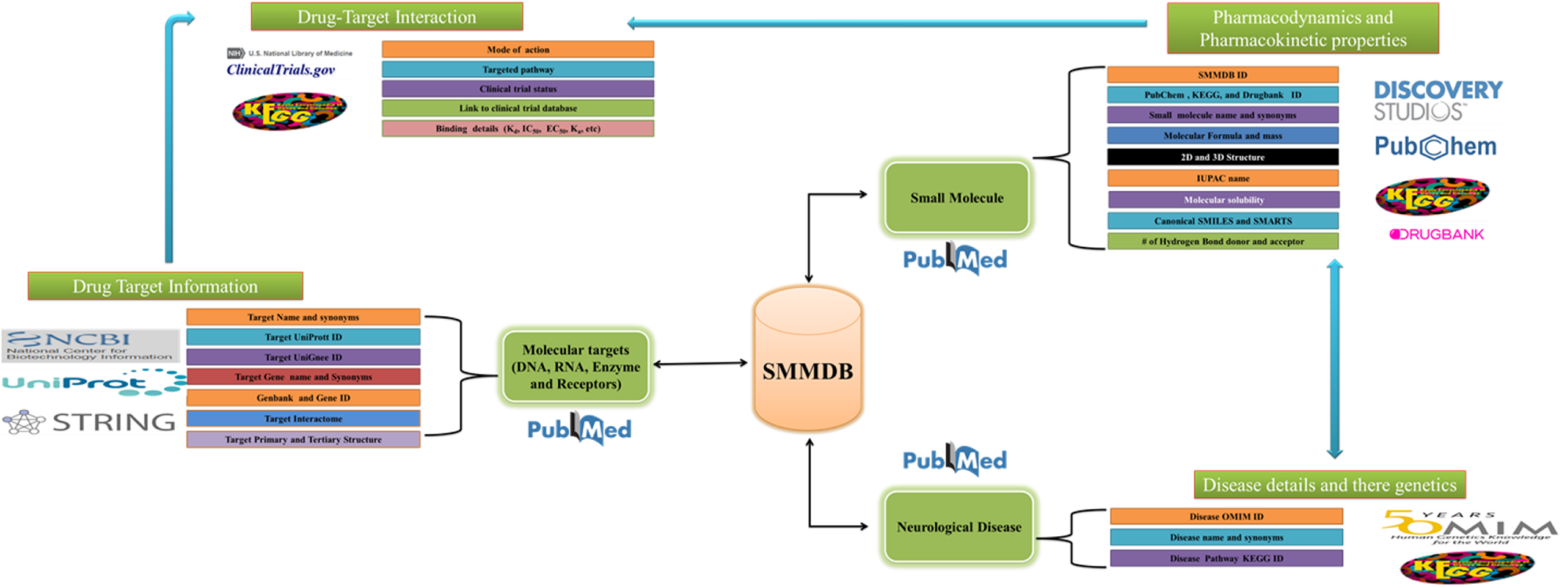
Organization of SMMDB. Schematic representation of SMMDB architecture.

The GUI of SMMDB database is available at the web URL http://bsbe.iiti.ac.in/bsbe/SMMDB/index.php. Figure 1 depicts the screenshot of SMMDB home page illustrating browse, search and download options. A schematic diagram illustrating its architecture and data collection protocol is shown in Figure 2. Browse option contains the list of small molecule modulators and provide various information such as SMMDB ID, their common name and synonyms, 2D structure, disease name and there OMIM identifier, respective Pubmed ID, current clinical trial status, link for the clinical trial database to avail the more information about the clinical studies, PubChem Compound, small molecule target name and mode of action. SMMDB ID is the unique identifier given to each molecule that is available in the database. SMMDB ID is hyperlinked with another page that contains the detailed pharmacokinetic and pharmacodynamics properties of the small molecule modulators such as binding affinity of small molecule with molecular targets (DNA, RNA or Protein), target name and their UniProt, UniGene and Genbank ID, 3D structure, SMILE and SMARTS notation, chemical and IUPAC name, International Chemical Identifier, LogD, molecular formula, molecular mass, molecular solubility, molecular weight, pKa, hydrogen bond acceptor count, hydrogen bond donor count, number of aromatic rings, number of chains, number of rotatable bonds, molecular polar surface area, molecular surface area, dipole magnitude, average bond length, minimized energy, radius of gyration and strain energy. GUI of SMMDB allows the users to perform query search in the form of text or structure and advanced searches that are based on some parameter. It further allows downloading of the 3D conformers for the all the small molecules available in SMMDB.

### Tools to search the database efficiently

SMMDB provides various web-based search tool to reduce the time cost of database users and get the useful information in minimum clicks.

### Quick search option

To explore the SMMDB database content rapidly, we have provided quick search option on the home page of SMMDB. Users can acquire results by employing any keywords like a common name, chemical name or IUPAC name of small molecules, disease name, disease OMIM ID, target name etc.

### Advance search option

**Figure 3:**
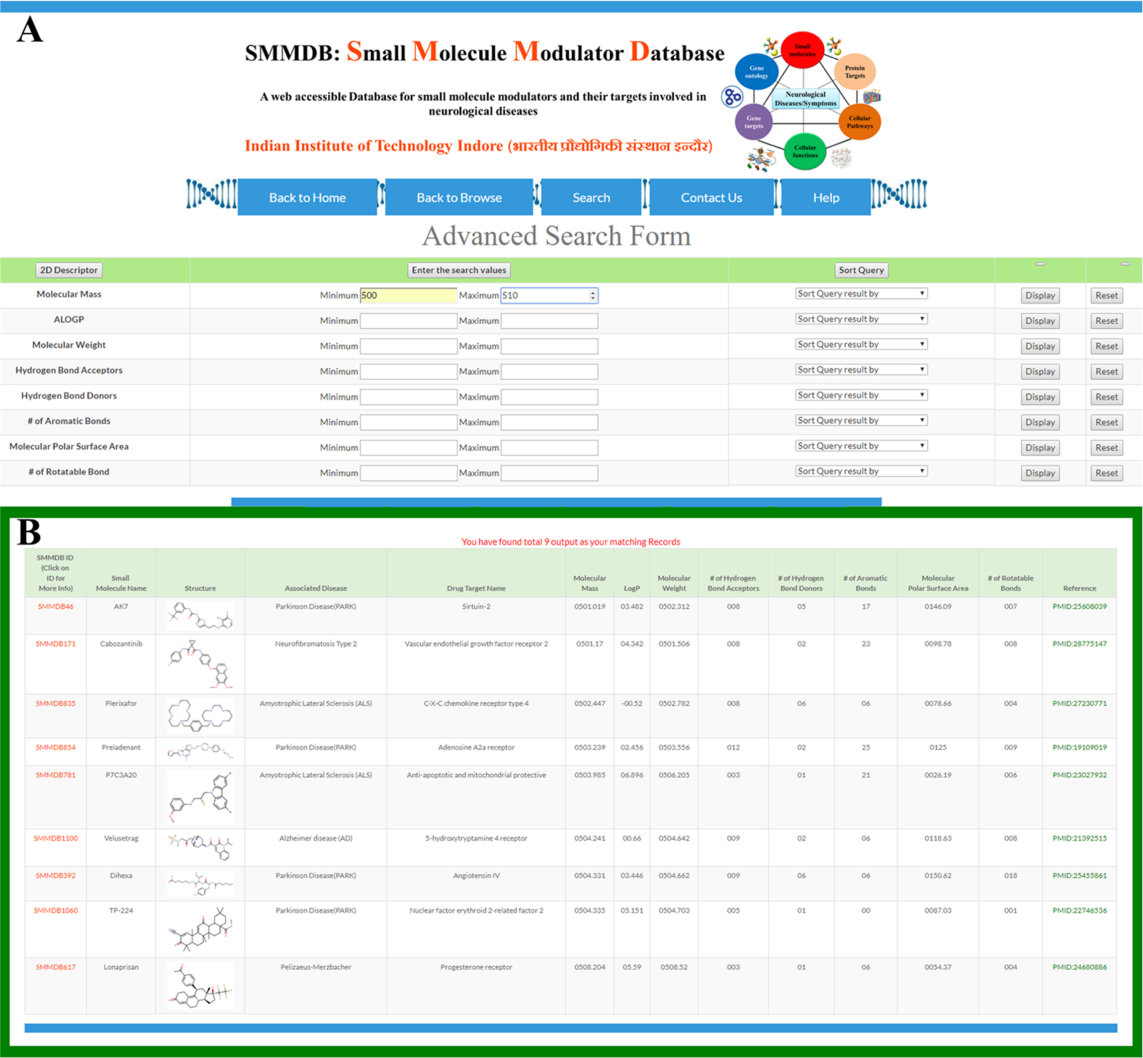
Advanced search. Screenshot showing **(A)** advanced search form and **(B)** an example of output of advanced search option.

We have integrated an advanced search tool to search the database molecules on the basis of their drug-likeness properties (Figure 3A) that are defined by Lipinski’s Rule of Five. The output of this advanced search option would provide the information of molecule that matches the user given search parameter (38). This will facilitate the initial screening of small molecule based on the specific property or a set of properties. An illustration for the input query and output of advanced search tool has been shown in Figure 3A and B.

### Sub-structure search tool

**Figure 4:**
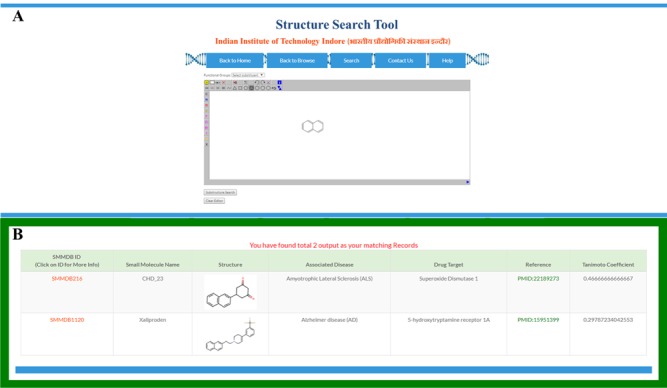
Structural search. Screen shot of **(A)** structure search tool and **(B)** output of structure search form.

To ease the searching of small molecules on the basis of their molecular structure, we embedded a structure search tool in the SMMDB (Figure 4A). This tool allows the searching of database small molecules by their similarity or identity with the structure of query molecule. Query molecule could be entered either by drawing its structure using JSME-based structure editor platform (39) or uploading the query molecule in various supported file formats. JSME is a Java applet-dependent cross-platform that allows the drawing, editing and displaying of small molecule within the webpage. The uploaded or drawn query molecule is immediately converted to their fingerprint pattern and temporarily stored as sub-graph and mine the available graph of the reference molecules that are already stored in database.

The searching of sub-graph to the reference graph is the key step to structure search algorithm. A graph is an embrace of set of vertices and arcs. Vertices are the fundamental unit of graphs and arcs are ordered pairs of nodes that have some associated costs. The structure search algorithm consider the graph of query and database molecule as two different argument and establish a correlation between two pairs on the basis of the mathematical construct of tanimoto coefficient. This tanimoto coefficient quantifies the similarity between two pairs using equation (1).

(1) \begin{equation*} \mathrm{Tanimoto}\ \mathrm{coefficient}=1-\frac{\mathrm{Na}\ \&\ b}{\mathrm{Na}+\mathrm{Nb}-\mathrm{Na}\&b}\kern0.5em \end{equation*}

where Na and Nb are the numbers of bits set present in the fingerprint of query and reference molecule, respectively, and Na&b is the number of bits set that are present in fingerprint of both query and reference molecule. The valid value of tanimoto coefficient can be any float value between 0 and 1, the ‘0’ value of the tanimoto score corresponds to no similarity between two structures while tanimoto score of ‘1’ suggests 100% similarity between query and reference molecule. Figure 4A and B depicting the example of query structure (two conjugated Benzene ring i.e. Naphthalene) designed in structure search tool and output of the substructure search result, respectively.

### Search the interaction network for the single or multiple drug targets

Currently, advancement in the understanding of cell signaling and pathways studies revealed novel and very effective drug targets that can be utilized to design a promising drug. Therefore, it becomes very important to know the interacting protein partners of a particular drug target or the interaction relationship between two drug targets for the same disease, for discovering the effective therapeutics. Using the API services available at the STRING database, we have embedded the ‘target interactome’ feature in SMMDB. Using this feature a user can get the details about the interaction network of single or multiple drug targets for a single disease.

### 2D and 3D structure visualization of ligands

**Figure 5:**
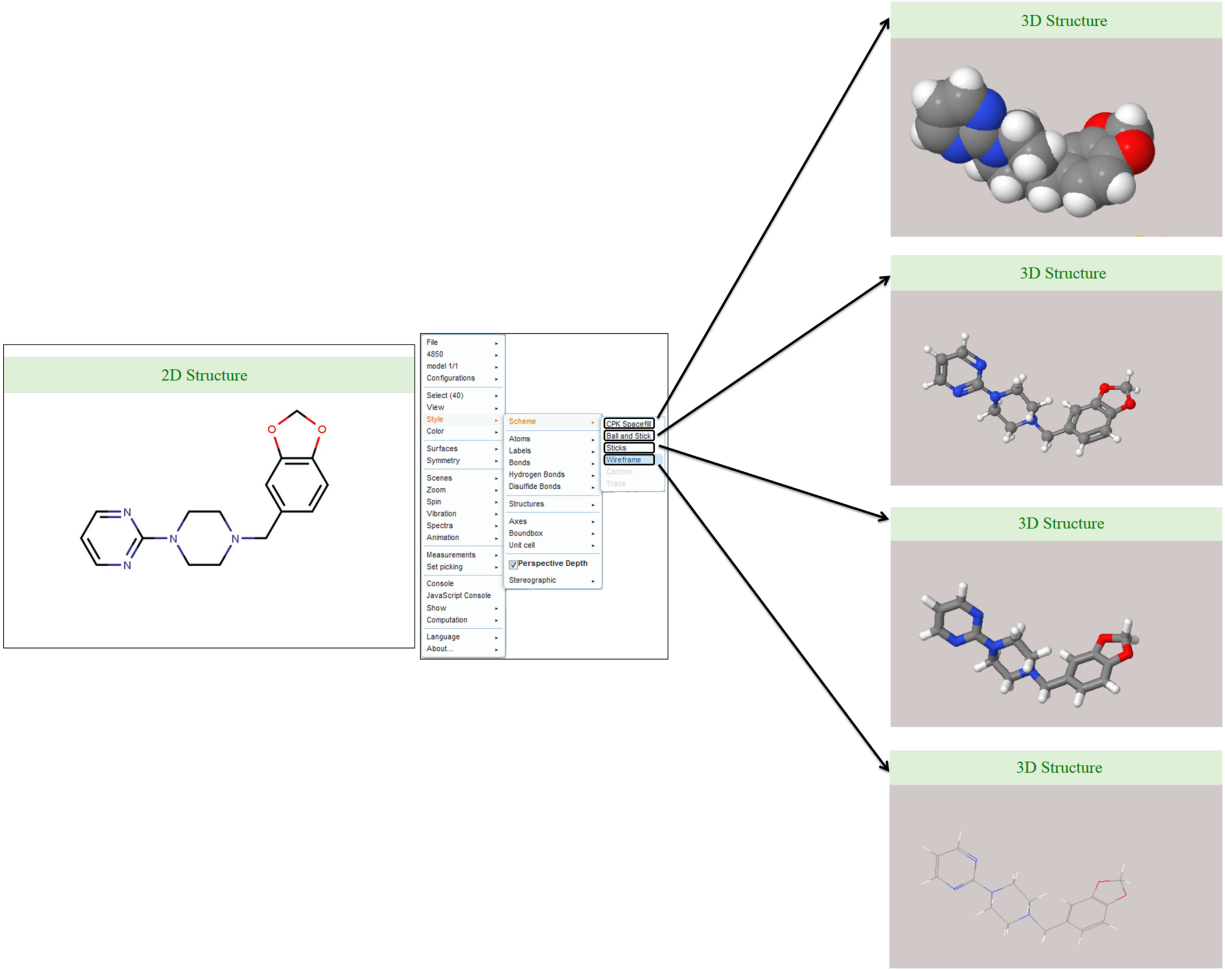
Ligand structure visualization. Screenshots of example showing various 2D and 3D structure representations.

Discovery Studio 4.0 was used for the 2D structure generation of ligand/drug molecule and saved as .jpeg image file format. The 3D coordinates of each ligand/drug molecule are generated in Discovery Studio 4.0 and can be visualized in the database using JSME-based Java applet. The default display is set to the ball-and-stick model with CPK scheme that can be further changed in another model according to the choice of database users such as wireframe, wireframe with knobs and space fill model (Figure 5).

### Download option for 3D conformation of database ligands

We have used Discovery Studio 4.0 to generate the 3D conformations of all database molecules and provide the option to download all the conformations in a single file. A maximum of 360 conformers were generated for each ligand present in SMMDB database (parameter for conformer generation files are described in Supplementary Table S5). These 3D conformations of small molecule may facilitate the drug discovery process and lead optimization task by involving in the shape-based virtual screening process (40–43).

#### Application of Study

To showcase how the SMMDB is capable of fastening up the drug discovery process specifically for neurological disease, we will use the SMMDB’s data for two neurological diseases namely (i) ALS/frontotemporal dementia (ALS/FTD) and (ii) Fragile X-Associated Tremor Ataxia Syndrome/FXS (FXTAS/FXS).

ALS/FTD and FXTAS are the two neurological disorders that have currently no effective treatment. ALS/FTD involved the progressive loss of motor neurons or degeneration of cortex of frontal and temporal complex that caused the cognitive and behavioral impairment in the patient (44). The expansion of the tandem repeats of r(GGGGCC) nucleotides in the c9orf72 gene affect the more than 40% cases of familial ALS/FTD and 10% cases of sporadic ALS/FTD (45). Mutation in the SOD gene is another major cause of both familial and sporadic ALS/FTD (46). FXTAS/FXS is also an inherited and unrecoverable neurodegenerative disorder caused by the expansion of tandem repeats of the r(CGG)exp repeats in the 5′ untranslated region (5′UTR) of the Fragile X mental retardation 1 (FMR1) gene, code for fragile X mental retardation protein (FMRP) (47).

### Search for SMMDB records regarding ALS/FTD and FXTAS

To begin, a query is initiated for all SMMDB’s records related to ALS/FTD and FXTAS disease. The disease-specific query returned 64 published studies that illustrated the total 135 kinds of small molecule/drugs and 43 types of drug targets for ALS/FTD disease. Whereas, a search on SMMDB for FXA/FXTAS disease, 29 published literature were returned that illustrated the total 37 small molecule/drug and 24 kinds of drug target for FXTAS/FXS disease (Supplementary File S2).

**Figure 6:**
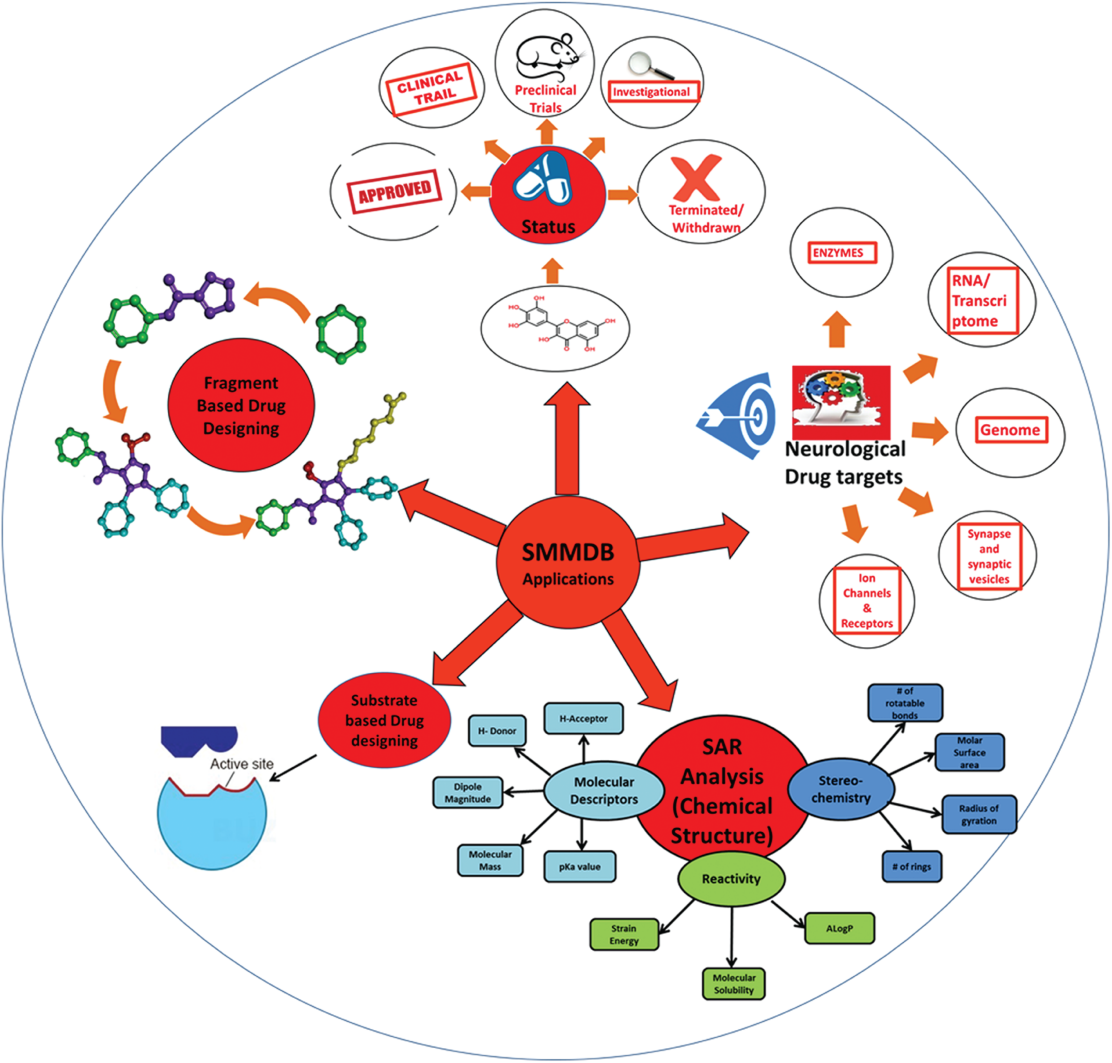
Application of SMMDB. Schematic representation for the various applications of SMMDB.

### Overlapping small molecules/drugs and drug targets calculation

The visual inspection of obtained data, allowed SMMDB’s users to obtain a multi-order demonstration (common small molecule/drugs and drug target) of disease data. This analysis revealed total three small molecules that are under clinical trial/investigation for both ALS/FTD and FXA/FXTAS disease (Supplementary Table S6). It is anticipated that a small molecule/drug selective for some particular molecular target in particular disease may investigate for the other disease as well that involved the same molecular drug targets as a reason for pathogenesis. This overlapping drug target analysis demonstrated that information available on SMMDB database could be helpful in analyzing the overlapping drug target analysis and quickly suggesting promising drug candidate for the uncured neurological disease. For example, Disney *et al.* (48) in 2012 successfully investigated a small molecule 1a (NSC311153) for the treatment of FXTAS by targeting the expanded tandem repeats of r(CGG)n nucleotides in fmr1 gene. However, it took two years to publish the same molecule for the treatment of ALS/FTD by targeting similar kind tandem repeat, i.e., r(GGGGCC)n in c9orf72 gene (49). Therefore, similar overlapping analysis for another set of diseases may explore novel small molecule or drug targets that can be repurposed in other unexplored neurological diseased condition and may save the precious time of scientific community. The workflow that was followed to explore for the enrichment analysis of SMMDB’s data in a more integral manner suggested the biological importance of SMMDB in drug discovery process for the neurological disease.

## Discussion

Within last few decades, the advent of small molecule modulators became the foundational pillars in the drug discovery process that have comparable advantage of drugability such as higher stability, absorption at the target site in comparison to small peptide and si-RNA-based therapeutic approaches. Because of these benefits, engineered small molecules modulators have provided the hope of promising clinical benefits and also explore the understanding of the cellular signaling pathways and microenvironment.

We have built SMMDB database with an attempt to assist the scientific community by providing comprehensive details about such engineered small molecule or small molecule modulators of natural origin that have been tested for their therapeutic potential for combating the neurological disorders. Here, we have covered ∼700 unique small molecule modulators that are reported to be involved in 122 neurological disorders by targeting ~∼300 molecular targets by the reviewing of >3000 research articles.

Very interestingly SMMDB contains the information of ~125 small molecules that are under investigation and their structures are not available in any other drug database. The database also contains a list of drugs that were in clinical trials but are now terminated or withdrawn due to failure or some other reasons. Researchers engaged in therapeutic development for a different neurological disease could efficiently use the information assembled here on a single source. It would ease SAR analysis, shape-based virtual screening, molecular docking studies, statistical studies and fragment-based drug discovery process. Figure 6 depicts the possible outcomes and application of the SMMDB database. Further, SMMDB will be routinely updated twice in a year to include more updated entries in the database. In this way, the scope of this database majorly covers the interest of scientific community and researchers who are continually putting their endeavor toward the study of pathogenic mechanism and development of more potent therapeutic compounds for various kind of neurological disorders. We are continually searching the updated scientific article and patent to make database up to date and include more experimentally validated data. We believe that the comprehensive set of data and efficient browsing tool of SMMDB would gravitate the attention of the scientific community from various backgrounds and fasten the drug development process for the neurological disorders.

## Supplementary Material

Supplementary Data

## Notes

**Database URL**: http://bsbe.iiti.ac.in/bsbe/smmdb
