# Supplementary material for: SMMDB: a web-accessible database for small molecule modulators and their targets involved in neurological diseases
Source: Database (Oxford). 2018 Sep 13;2018:bay082. doi: 10.1093/database/bay082 (PMC6146116; doi:10.1093/database/bay082)
Supplement: Supplementary Data [file bay082_supp.zip › Supplementary file S1.pdf]

Supplementary Information S1

SMMDB: a web accessible Database for small molecule modulators and their targets involved in neurological diseases

Subodh Kumar Mishra[1], Neha Jain[1], Uma Shankar[1], Arpita Tawani[1], Amit Mishra[2], Amit Kumar[1,*]

[1] Discipline of Biosciences and Biomedical Engineering, Indian Institute of Technology Indore, Indore, Simrol, 453552, India
[2]Cellular and Molecular Neurobiology Unit, Indian Institute of Technology Jodhpur, Rajasthan 342011, India

*Correspondence author: Tel: +91-731-2438771; Fax: +91-731-2364182; Email: amitk@iiti.ac.in

Supplementary Table S1: Complete list of small molecule and their target that have been studied for their therapeutic potential in case of amyotrophic lateral sclerosis (ALS).

Drug Name	Status	Pubmed ID	Drug Target Name	Uniprot ID of Target
Compound_23	Under Investigation	22922411	Ephrin type-A receptor 4	P54764
052C9	Under Investigation	18627324	Nuclear factor erythroid 2-related factor 2	Q16236
14-25772747	Under Investigation	25772747	Superoxide Dismutase 1	P00441
AGS-499	Under Investigation	22351600	Telomerase	O14746
AR-A014418	Under Investigation	17433298	Glycogen synthase kinase-3 beta	P49841
Arimoclomol	Phase II/III	23978556	Superoxide Dismutase 1	P00441
Bromocriptine	Under Investigation	21867702	D(2) dopamine receptor	P14416
Calpastatin	Under Investigation	26756888	Nuclear factor erythroid 2-related factor 2	Q16236
CDDO-EA	Under Investigation	21457778	Nuclear factor erythroid 2-related factor 2	Q16236
CDDO-TFEA	Under Investigation	21457778	Nuclear factor erythroid 2-related factor 2	Q16236
Ceftriaxone	Clinical Trail Completed	25297012	Glutamine synthetase	P15104
CHD_10	Under Investigation	22189273	Superoxide Dismutase 1	P00441
CHD_11	Under Investigation	22189273	Superoxide Dismutase 1	P00441
CHD_12	Under Investigation	22189273	Superoxide Dismutase 1	P00441
CHD_13	Under Investigation	22189273	Superoxide Dismutase 1	P00441
CHD_14	Under Investigation	22189273	Superoxide Dismutase 1	P00441
CHD_15	Under Investigation	22189273	Superoxide Dismutase 1	P00441
CHD_16	Under Investigation	22189273	Superoxide Dismutase 1	P00441
CHD_17	Under Investigation	22189273	Superoxide Dismutase 1	P00441
CHD_18	Under Investigation	22189273	Superoxide Dismutase 1	P00441
CHD_19	Under Investigation	22189273	Superoxide Dismutase 1	P00441
CHD_20	Under Investigation	22189273	Superoxide Dismutase 1	P00441
CHD_21	Under Investigation	22189273	Superoxide Dismutase 1	P00441
CHD_22	Under Investigation	22189273	Superoxide Dismutase 1	P00441
CHD_23	Under Investigation	22189273	Superoxide Dismutase 1	P00441
CHD_24	Under Investigation	22189273	Superoxide Dismutase 1	P00441
CHD_25	Under Investigation	22189273	Superoxide Dismutase 1	P00441
CHD_26	Under Investigation	22189273	Superoxide Dismutase 1	P00441
CHD_27	Under Investigation	22189273	Superoxide Dismutase 1	P00441
CHD_28	Under Investigation	22189273	Superoxide Dismutase 1	P00441
CHD_29	Under Investigation	22189273	Superoxide Dismutase 1	P00441
CHD_30	Under Investigation	22189273	Superoxide Dismutase 1	P00441
CHD_31	Under Investigation	22189273	Superoxide Dismutase 1	P00441
CHD_32	Under Investigation	22189273	Superoxide Dismutase 1	P00441
CHD_33	Under Investigation	22189273	Superoxide Dismutase 1	P00441
CHD_34	Under Investigation	22189273	Superoxide Dismutase 1	P00441
CHD_35	Under Investigation	22189273	Superoxide Dismutase 1	P00441
CHD_36	Under Investigation	22189273	Superoxide Dismutase 1	P00441

CHD_37	Under Investigation	22189273	Superoxide Dismutase 1	P00441
CHD_38	Under Investigation	22189273	Superoxide Dismutase 1	P00441
CHD_39	Under Investigation	22189273	Superoxide Dismutase 1	P00441
CHD_40	Under Investigation	22189273	Superoxide Dismutase 1	P00441
CHD_41	Under Investigation	22189273	Superoxide Dismutase 1	P00441
CHD_42	Under Investigation	22189273	Superoxide Dismutase 1	P00441
CHD_43	Under Investigation	22189273	Superoxide Dismutase 1	P00441
CHD_44	Under Investigation	22189273	Superoxide Dismutase 1	P00441
CHD_45	Under Investigation	22189273	Superoxide Dismutase 1	P00441
CHD_46	Under Investigation	22189273	Superoxide Dismutase 1	P00441
CHD_47	Under Investigation	22189273	Superoxide Dismutase 1	P00441
CHD_48	Under Investigation	22189273	Superoxide Dismutase 1	P00441
CHD_49	Under Investigation	22189273	Superoxide Dismutase 1	P00441
CHD_50	Under Investigation	22189273	Superoxide Dismutase 1	P00441
CHD_51	Under Investigation	22189273	Superoxide Dismutase 1	P00441
CHD_52	Under Investigation	22189273	Superoxide Dismutase 1	P00441
CHD_53	Under Investigation	22189273	Superoxide Dismutase 1	P00441
CHD_54	Under Investigation	22189273	Superoxide Dismutase 1	P00441
CHD_55	Under Investigation	22189273	Superoxide Dismutase 1	P00441
CHD_56	Under Investigation	22189273	Superoxide Dismutase 1	P00441
CHD_57	Under Investigation	22189273	Superoxide Dismutase 1	P00441
CHD_58	Under Investigation	22189273	Superoxide Dismutase 1	P00441
CHD_59	Under Investigation	22189273	Superoxide Dismutase 1	P00441
CHD_60	Under Investigation	22189273	Superoxide Dismutase 1	P00441
CHD_61	Under Investigation	22189273	Superoxide Dismutase 1	P00441
CHD_62	Under Investigation	22189273	Superoxide Dismutase 1	P00441
CHD_63	Under Investigation	22189273	Superoxide Dismutase 1	P00441
CHD_64	Under Investigation	22189273	Superoxide Dismutase 1	P00441
CHD_65	Under Investigation	22189273	Superoxide Dismutase 1	P00441
CHD_66	Under Investigation	22189273	Superoxide Dismutase 1	P00441
CHD_67	Under Investigation	22189273	Superoxide Dismutase 1	P00441
CHD_68	Under Investigation	22189273	Superoxide Dismutase 1	P00441
CHD_69	Under Investigation	22189273	Superoxide Dismutase 1	P00441
CHD_7	Under Investigation	22189273	Superoxide Dismutase 1	P00441
CHD_70	Under Investigation	22189273	Superoxide Dismutase 1	P00441
CHD_71	Under Investigation	22189273	Superoxide Dismutase 1	P00441
CHD_72	Under Investigation	22189273	Superoxide Dismutase 1	P00441
CHD_73	Under Investigation	22189273	Superoxide Dismutase 1	P00441
CHD_8	Under Investigation	22189273	Superoxide Dismutase 1	P00441
CHD_9	Under Investigation	22189273	Superoxide Dismutase 1	P00441
Compound ID 7687685	Under Investigation	22652679	Superoxide Dismutase 1	P00441
CPN-9	Under Investigation	23000247	Nuclear factor erythroid 2-related factor 2	Q16236
Creatine	Phase II Completed	28980128	Creatine kinase M-type	P06732
Cu(II)ATSM	Phase I/II	24899723	Superoxide Dismutase 1	P00441
DB1246	Under Investigation	29113975	r(GGGGCC)exp C9ORF72 RNA	Q96LT7
DB1247	Under Investigation	29113975	r(GGGGCC)exp C9ORF72 RNA	Q96LT7

DB1273	Under Investigation	29113975	r(GGGGCC)exp C9ORF72 RNA	Q96LT7
Dehydroepiandrosterone	Terminated in Preclincal trail	22409357	Androgen receptor	P10275
Dexpramipexole	Terminated at Phase III	22985432	D(2) dopamine receptor	P14416
Dichloroacetate	Under Investigation	22509356	Pyruvate dehydrogenase [lipoamide] kinase isozyme 1, mitochondrial	Q15118
Dihydrotestosterone	Under Investigation	22606355	Androgen receptor	P10275
DL-3-n-butylphthalide	Under Investigation	22056419	Nuclear factor erythroid 2-related factor 2	Q16236
Edaravone	Phase III	28872919	potent scavenger of oxygen radicals	Not Available
Edaravone	Phase III Completed	18718468	Free radical scavenger	Not Available
ER-000444793	Under investigation	25565966	Mitochondrial permeability transition pore (mPTP)	Not Available
Ezogabine	Phase II	27314534	Potassium voltage-gated channel subfamily KQT member 2	O43526
Fasudil	Phase II	28197100	Rho-associated protein kinase (ROCK)	Q13464
Fingolimod	Phase II	28662296	Sphingosine-1 phosphate receptor 5	Q9H228
Gapmers – ASO	Phase I	24139042	r(GGGGCC)exp C9ORF72 RNA	Q96LT7
Ibudilast	Phase II	26005122	cAMP-specific 3',5'-cyclic phosphodiesterase 4A	P27815
ISIS-SOD1RX	Phase I	23541756	Superoxide Dismutase 1	P00441
ISIS-SOD1RX	Phase I Completed	23541756	Superoxide Dismutase 1	P00441
Kenpaullone	Under Preclincal trail	23602540	Glycogen synthase kinase-3 beta	P49841
L-745870	Under Investigation	18423451	D(4) dopamine receptor	P21917
Lithium Carbonate	Phase II	24667005	Glutamate receptor 3	P42263
Masitinib	Phase II/III Completed	27400786	Tyrosine-protein kinase ABL1	P00519
Mecobalamin	Phase II/III Completed	25982504	Not Available	Not Available
Memantine	Phase II	24356417	Glutamate receptor ionotropic, NMDA 3A	Q8TCU5
Mexiletine	Phase II	26911633	Sodium channel protein type 5 subunit alpha	Q14524
Miglustat	Under Investigation	28229532	Ceramide glucosyltransferase	Q16739
NP001	Phase II	25884010	inflammatory macrophages and monocytes	Not Available
NSC311153 (1a)	Under Investigation	25132468	r(GGGGCC)exp C9ORF72 RNA	Q96LT7
NSC377363	Under trial	25132468	r(GGGGCC)exp C9ORF72 RNA	Q96LT7
NSC699145	Under trial	25132468	r(GGGGCC)exp C9ORF72 RNA	Q96LT7
Nuedexta	Phase II Completed	28283968	Sigma non-opioid intracellular receptor 1	Q99720
Olesoxime	Phase II/III Completed	20721828	Mitochondrial permeability transition pore (mPTP)	Not Available
P7C3A20	Under Investigation	23027932	Anti-apoptotic and mitochondrial protective	Not Available
Perampanel	Phase I/II	27350567	Glutamate receptor 1	P42261
Pimozide	Phase II	9864711	D(2) dopamine receptor	P14416
Pimozide	Phase II	9864711	D(2) dopamine receptor	P14416
Pioglitazone	Terminated at Phase II	22715372	Peroxisome proliferator-activated receptor gamma	P37231
Plerixafor	Under Investigation	27230771	C-X-C chemokine receptor type 4	P61073
Procysteine	Under Investigation	10227642	Reactive oxygen species	Not Available

Pyrimethamine	Phase I/II Completed	28480639	Superoxide Dismutase 1	P00441
Quercetin 3-beta-D-glucoside	Under Investigation	28475686	Superoxide Dismutase 1	P00441
Quercitrin	Under Investigation	28475686	Superoxide Dismutase 1	P00441
Rasagiline	Phase II	15372249	Amine oxidase [flavin-containing] B	P27338
Riluzole	FDA approved	22892214	Metabotropic glutamate receptor 1	Q13255
Riluzole	FDA approved	22892214	Sodium channel protein type 5 subunit alpha	Q14524
Sodium phenylbutyrate	Phase I/II Completed	15934930	Histone deacetylase 1	Q13547
SUN N8075	Under Investigation	21151573	Neurosecretory protein VGF	O15240
Talampanel	Terminated at Phase II	21623665	Metabotropic glutamate receptor 2	Q14416
Talampanel	Terminated at Phase II	21623665	Glutamate receptor 1	P42261
Tauroursodeoxycholic acid (TUDCA)	Phase II	25664595	Solute carrier organic anion transporter family member 1A2	P46721
Tauroursodeoxycholic acid (TUDCA)	Phase II/III	25664595	Solute carrier organic anion transporter family member 1A2	P46721
Thalidomide	Terminated at Phase II	19922130	Prostaglandin G/H synthase 2	P35354
Tirasemtiv	PhaseII/III	26982815	Troponin	P07437
TMPyP4 Porphyrin	Under Investigation	24371143	r(GGGGCC)exp C9ORF72 RNA	Q96LT7
Trichostatin A	Under Investigation	21712032	Histone deacetylase 1	Q13547
Vardenafil	Under Investigation	29113829	Insulin-like growth factor II	P01344

Supplementary Table S2: List of keywords used for searching in PubMed and number of hits obtained for each.

Keywords used in NCBI PubMed Database	Number of hits	Positive hits	Miss-matching cases*
Amyotrophic Lateral Sclerosis			
Amyotrophic Lateral Sclerosis (ALS) AND Small molecule	104	10	94
Amyotrophic Lateral Sclerosis (ALS) AND drug	2354	50	2304
ALS AND drug	3370	52	3318
Approved drugs for ALS	58	1	57
small molecules under investigation for ALS	2	0	2
ALS AND therapeutics	3144	13	3131
Parkinson Disease			
Parkinson Disease AND Small molecule	190	30	160
Parkinson Disease AND drug	25444	135	25309
Approved drugs for Parkinson Disease	109	1	108
small molecules under investigation for Parkinson Disease	1	0	1
Parkinson Disease AND therapeutics	15297	36	15261
Alzheimer disease (AD)			
Alzheimer disease (AD) AND Small molecule	546	19	527
Alzheimer disease (AD) AND drug	23228	123	23105
Approved drugs for Alzheimer disease (AD)	364	3	361
Small molecules under investigation for Alzheimer disease (AD)	5	1	4
Alzheimer disease (AD) AND therapeutics	12246	32	12214
Epilepsy			
Epilepsy AND Small molecule	109	4	105
Epilepsy AND drug	45700	41	45659

Approved drugs for Epilepsy	489	3	486
Small molecules under investigation for Epilepsy	0	0	0
Epilepsy AND therapeutics	19868	7	19861
Huntington Disease (HD)			
Huntington Disease (HD) AND Small molecule	38	4	34
Huntington Disease (HD) AND drug	1236	42	1194
Approved drugs for Huntington Disease (HD)	10	0	10
Small molecules under investigation for Huntington Disease (HD)	1	0	1
Huntington Disease AND therapeutics	1917	19	1898
Migraine			
Migraine AND Small molecule	63	0	63
Migraine AND drug	11991	40	11951
Approved drugs for Migraine	152	3	149
Small molecules under investigation for Migraine	0	0	0
Migraine AND therapeutics	5641	16	5625
Schizophrenia			
Schizophrenia AND Small molecule	135	0	135
Schizophrenia AND drug	42098	53	42045
Approved drugs for Schizophrenia	254	4	250
Small molecules under investigation for Schizophrenia	1	1	0
Schizophrenia AND therapeutics	23126	18	23108
Tardive Dyskinesia			
Tardive Dyskinesia AND Small molecule	2	0	2
Tardive Dyskinesia AND drug	3919	15	3904
Approved drugs for Tardive Dyskinesia	18	2	16
Small molecules under investigation for Tardive Dyskinesia	0	0	0
Tardive Dyskinesia AND therapeutics	789	5	784
Wilson Disease			
Wilson Disease AND Small molecule	89	0	89
Wilson Disease AND drug	5079	5	5074
Approved drugs for Wilson Disease	21	0	21
Small molecules under investigation for Wilson Disease	0	0	0
Wilson Disease AND therapeutics	4403	2	4401
Traumatic brain injuries			
Traumatic brain injuries AND Small molecule	46		46
Traumatic brain injuries AND drug	4632	17	4615
Approved drugs for Traumatic brain injuries	24	1	23
Small molecules under investigation Traumatic brain injuries	1	0	1
Traumatic brain injuries AND therapeutics	6856	6	6850
Spinal Muscular Atrophy			
Spinal Muscular Atrophy AND Small molecule	53	3	50
Spinal Muscular Atrophy AND drug	729	9	720
Approved drugs for spinal Muscular Atrophy	14	1	13

Small molecules under investigation Spinal Muscular Atrophy	0	0	0
Spinal Muscular Atrophy AND therapeutics	979	1	978
Spinocerebellar Ataxia			
Spinocerebellar Ataxia AND Small molecule	24	1	23
Spinocerebellar Ataxia AND drug	965	6	959
Approved drugs for Spinocerebellar Ataxia	3	0	3
Small molecules under investigation Spinocerebellar Ataxia	0	0	0
Spinocerebellar Ataxia AND therapeutics	454	1	453
Neurofibromatosis			
Neurofibromatosis AND Small molecule	30	0	30
Neurofibromatosis AND drug	983	11	972
Approved drugs for Neurofibromatosis	7	0	7
Small molecules under investigation Neurofibromatosis	0	0	0
Neurofibromatosis AND therapeutics	1372	3	1369
Multiple Sclerosis			
Multiple Sclerosis AND Small molecule	482	2	480
Multiple Sclerosis AND drug	19039	33	19006
Approved drugs for Multiple Sclerosis	468	2	466
Small molecules under investigation Multiple Sclerosis	4	0	4
Multiple Sclerosis AND therapeutics	12442	14	12428
Restless legs syndrome			
Restless legs syndrome AND Small molecule	0	0	0
Restless legs syndrome AND drug	1520	6	1514
Approved drugs for restless legs syndrome	25	0	25
Small molecules under investigation Restless legs syndrome	0	0	0
Restless legs syndrome AND therapeutics	690	3	687
Fragile X Syndrome			
Fragile X Syndrome AND Small molecule	29	6	23
Fragile X Syndrome AND drug	632	25	607
Approved drugs for Fragile X Syndrome	9	2	7
Small molecules under investigation AND Fragile X Syndrome	0	0	0
Fragile X Syndrome AND therapeutics	310	8	302
Essential Tremor			
Essential Tremor AND Small molecule	1	0	1
Essential Tremor AND drug	870	29	841
Approved drugs for Essential Tremor	4	0	4
Small molecules under investigation AND essential Tremor	0	0	0
Essential Tremor AND therapeutics	912	11	901
Complex Regional Pain Syndrome			
complex Regional Pain Syndrome AND Small molecule	1	0	1
complex Regional Pain Syndrome AND drug	1173	6	1167
Approved drugs for complex Regional Pain Syndrome	3	0	3
Small molecules under investigation AND complex Regional Pain Syndrome	0	0	0

complex Regional Pain Syndrome AND therapeutics	1704	4	1700
Prion disease			
Prion disease AND Small molecule	113	1	112
Prion disease AND drug	2212	1	2211
Approved drugs for Prion disease	11	0	11
Small molecules under investigation AND Prion disease	2	0	2
Prion disease AND therapeutics	1430	2	1428
Bipolar disorder			
Bipolar disorder AND Small molecule	26	0	26
Bipolar disorder AND drug	17869	13	17856
Approved drugs for Bipolar disorder	179	0	179
Small molecules under investigation Bipolar disorder	0	0	0
Bipolar disorder AND therapeutics	8849	5	8844
Carpal Tunnel Syndrome			
Carpal Tunnel Syndrome AND Small molecule	3	0	3
Carpal Tunnel Syndrome AND drug	681	6	675
Approved drugs for Carpal Tunnel Syndrome	0	0	0
Small molecules under investigation AND Carpal Tunnel Syndrome	0	0	0
Carpal Tunnel Syndrome AND therapeutics	1683	5	1678
Familial Dysautonomia (FD)			
Familial Dysautonomia AND Small molecule	1	0	1
Familial Dysautonomia AND drug	272	4	268
Approved drugs for Familial Dysautonomia	2	0	2
Small molecules under investigation Familial Dysautonomia	0	0	0
Familial Dysautonomia AND therapeutics	129	1	128
Friedreich ataxia			
Friedreich ataxia AND Small molecule	12	0	12
Friedreich ataxia AND drug	343	10	333
Approved drugs for Friedreich ataxia	3	0	3
Small molecules under investigation AND Friedreich ataxia	0	0	0
Friedreich ataxia AND therapeutics	174	2	172
Frontotemporal Dementia			
Frontotemporal Dementia AND Small molecule	11	0	11
Frontotemporal Dementia AND drug	510	4	506
Approved drugs for Frontotemporal Dementia	12	0	12
Small molecules under investigation AND Frontotemporal Dementia	0	0	0
Frontotemporal Dementia AND therapeutics	378	1	377
Kleine-Levin Syndrome			
Kleine-Levin Syndrome AND Small molecule	0	0	0
Kleine-Levin Syndrome AND drug	96	7	89
Approved drugs for Kleine-Levin Syndrome	0	0	0
Small molecules under investigation AND Kleine-Levin Syndrome	0	0	0
Kleine-Levin Syndrome AND therapeutics	23	1	22

Narcolepsy			
Narcolepsy AND Small molecule	9	1	8
Narcolepsy AND drug	1639	7	1632
Approved drugs for Narcolepsy	50	1	49
Small molecules under investigation AND Narcolepsy	0	0	0
Narcolepsy AND therapeutics	584	4	580
Niemann-Pick Disease			
Niemann-Pick Disease AND Small molecule	28	1	27
Niemann-Pick Disease AND drug	632	5	627
Approved drugs for Niemann-Pick Disease	12	0	12
Small molecules under investigation AND Niemann-Pick Disease	0	0	0
Niemann-Pick Disease AND therapeutics	272	1	271
Seizures			
Seizures AND Small molecule	49	0	49
Seizures AND drug	43023	12	43011
Approved drugs for Seizures	370	0	370
Small molecules under investigation AND Seizures	0	0	0
Seizures AND therapeutics	6000	6	5994
Trichotillomania			
Trichotillomania AND Small molecule	0		0
Trichotillomania AND drug	248	5	243
Approved drugs for Trichotillomania	1	0	1
Small molecules under investigation AND Trichotillomania	0	0	0
Trichotillomania AND therapeutics	3	0	3
Tuberous Sclerosis			
Tuberous Sclerosis AND Small molecule	25	0	25
Tuberous Sclerosis AND drug	1305	5	1300
Approved drugs for Tuberous Sclerosis	15	0	15
Small molecules under investigation AND Tuberous Sclerosis	0	0	0
Tuberous Sclerosis AND therapeutics	661	0	661
Acute Ischemic Stroke			
Acute Ischemic Stroke AND Small molecule	26	0	26
Acute Ischemic Stroke AND drug	8357	1	8356
Approved drugs for Acute Ischemic Stroke	82	0	82
Small molecules under investigation AND Acute Ischemic Stroke	1	0	1
Acute Ischemic Stroke AND therapeutics	8764	3	8761
Adrenoleukodystrophy			
Adrenoleukodystrophy AND Small molecule	4	0	4
Adrenoleukodystrophy AND drug	305	2	303
Approved drugs for Adrenoleukodystrophy	0	0	0
Small molecules under investigation AND Adrenoleukodystrophy	0	0	0
Adrenoleukodystrophy AND therapeutics	287	0	287
Allan-Herndon-Dudley syndrome			

Allan-Herndon-Dudley syndrome AND Small molecule	0	0	0
Allan-Herndon-Dudley syndrome AND drug	16	1	15
Approved drugs for Allan-Herndon-Dudley syndrome	0	0	0
Small molecules under investigation AND Allan-Herndon-Dudley syndrome	0	0	0
Allan-Herndon-Dudley syndrome AND therapeutics	5	0	5
Alpers Disease			
Alpers Disease AND Small molecule	0	0	0
Alpers Disease AND drug	190	1	189
Approved drugs for Alpers Disease	0	0	0
Small molecules under investigation AND Alpers Disease	0	0	0
Alpers Disease AND therapeutics	9	0	9
Angelman syndrome			
Angelman syndrome AND Small molecule	4	0	4
Angelman syndrome AND drug	135	2	133
Approved drugs for Angelman syndrome	2	0	2
Small molecules under investigation AND Angelman syndrome	0	0	0
Angelman syndrome AND therapeutics	111	2	109
Aphasia			
Aphasia AND Small molecule	4	0	4
Aphasia AND drug	1039	2	1037
Approved drugs for Aphasia	3	0	3
Small molecules under investigation AND Aphasia	0	0	0
Aphasia AND therapeutics	2823	2	2821
Ataxia telangiectasia			
Ataxia telangiectasia AND Small molecule	125	0	125
Ataxia telangiectasia AND drug	2244	2	2242
Approved drugs for Ataxia telangiectasia	8	0	8
Small molecules under investigation AND Ataxia telangiectasia	0	0	0
Ataxia telangiectasia AND therapeutics	571	0	571
Attention Deficit Hyperactivity Disorder (ADHD)			
Attention Deficit Hyperactivity Disorder AND Small molecule	5	0	5
Attention Deficit Hyperactivity Disorder AND drug	9744	18	9726
Approved drugs for Attention Deficit Hyperactivity Disorder	139	0	139
Small molecules under investigation AND Attention Deficit Hyperactivity Disorder	0	0	0
Attention Deficit Hyperactivity Disorder AND therapeutics	4224	9	4215
Autism			
Autism AND Small molecule	57	0	57
Autism AND drug	4185	12	4173
Approved drugs for Autism	47	1	46
Small molecules under investigation AND Autism	0	0	0
Autism AND therapeutics	3876	7	3869
Charcot Marie Tooth disease (CMT)			
Charcot Marie Tooth disease AND Small molecule	11	0	11

Charcot Marie Tooth disease AND drug	272	2	270
Approved drugs for Charcot Marie Tooth disease	5	0	5
Small molecules under investigation AND Charcot Marie Tooth disease	0	0	0
Charcot Marie Tooth disease AND therapeutics	225	1	224
Coffin-Lowry syndrome			
Coffin-Lowry syndrome AND Small molecule	1	0	1
Coffin-Lowry syndrome AND drug	10	1	9
Approved drugs for Coffin-Lowry syndrome	0	0	0
Small molecules under investigation AND Coffin-Lowry syndrome	0	0	0
Coffin-Lowry syndrome AND therapeutics	7	0	7
Delayed Sleep Phase Syndrome			
Delayed Sleep Phase Syndrome AND Small molecule	4	0	4
Delayed Sleep Phase Syndrome AND drug	1398	3	1395
Approved drugs for Delayed Sleep Phase Syndrome	15	0	15
Small molecules under investigation AND Delayed Sleep Phase Syndrome	0	0	0
Delayed Sleep Phase Syndrome AND therapeutics	1126	3	1123

*** Also includes the repeated entries for the same disease – small molecule pair**

Supplementary Table S3: drugtargetinfo (stores the information about small molecule, disease and involved target)

**Field**	**Type**	**Null**	**Collation**	**Default**
Database ID	Int(100)	Yes	latin1_swedish_ci	None
Drug Name	Varchar(50)	No	latin1_swedish_ci	None
Drug Synonyms	Varchar(500)	Yes	latin1_swedish_ci	None
Status	Varchar(50)	No	latin1_swedish_ci	None
Disease Name	Varchar(50)	Yes	latin1_swedish_ci	None
Pubmed ID	Int(100)	No	latin1_swedish_ci	None
Disease Synonyms	Varchar(500)	Yes	latin1_swedish_ci	None
OMIM ID	Int(100)	No	latin1_swedish_ci	None
KEGG Drug ID	Int(100)	Yes	latin1_swedish_ci	None
Drug Bank ID	Int(100)	No	latin1_swedish_ci	None
Pubchem Compound ID	Int(100)	Yes	latin1_swedish_ci	None
IUPAC Name	Varchar(500)	Yes	latin1_swedish_ci	None
Drug Target Name	Varchar(500)	No	latin1_swedish_ci	None
Target Synonyms	Varchar(500)	Yes	latin1_swedish_ci	None
Pathway in which Target is involved	Varchar(500)	No	latin1_swedish_ci	None
Pathway Link	Varchar(500)	Yes	latin1_swedish_ci	None
Uniprot ID of Target	Varchar(500)	No	latin1_swedish_ci	None
UNIGENE	Varchar(500)	Yes	latin1_swedish_ci	None
Gene Name	Varchar(500)	Yes	latin1_swedish_ci	None

Gene Synonyms	Varchar(500)	No	latin1_swedish_ci	None
NCBI GENE ID	Varchar(500)	Yes	latin1_swedish_ci	None
Mode of Action	Varchar(500)	No	latin1_swedish_ci	None
KD	Varchar(500)	Yes	latin1_swedish_ci	None
REF	Varchar(500)	No	latin1_swedish_ci	None
EC50	Varchar(500)	Yes	latin1_swedish_ci	None
REF	Varchar(500)	No	latin1_swedish_ci	None
KI	Varchar(500)	Yes	latin1_swedish_ci	None
REFF	Varchar(500)	No	latin1_swedish_ci	None
IC50	Varchar(500)	Yes	latin1_swedish_ci	None
REF	Varchar(500)	No	latin1_swedish_ci	None
PDB	Varchar(500)	Yes	latin1_swedish_ci	None
Clinical Trials database	Varchar(500)	No	latin1_swedish_ci	None
STRID	Int (50)	Yes	latin1_swedish_ci	None

Where **PRI:** primary key, generally auto-generated by the database

UNI: this field will take only unique values

NULL: used to represent no value

varchar: MySQL data type, used for storing a string of limited length efficiently

int: MySQL data type, used to represent an integer

Supplementary Table S4: alldrug (stores information about pharmacokinetics and pharmacodynamics properties of small molecule)

Field	Type	Null	Collation	Default
Database ID	Int(100)	Yes	latin1_swedish_ci	None
STRID	Varchar(50)	No	latin1_swedish_ci	None
Pubchem Compound ID	Varchar(500)	Yes	latin1_swedish_ci	None
Canonical_Smiles	Varchar(50)	No	latin1_swedish_ci	None
ChemicalName	Varchar(50)	Yes	latin1_swedish_ci	None
Chime	Int(100)	No	latin1_swedish_ci	None
InChI	Varchar(500)	Yes	latin1_swedish_ci	None
InChI_AuxInfo	Int(100)	No	latin1_swedish_ci	None
InChIKey	Int(100)	Yes	latin1_swedish_ci	None
NemaKery	Int(100)	No	latin1_swedish_ci	None
SMARTS	Int(100)	Yes	latin1_swedish_ci	None
Smiles	Varchar(500)	Yes	latin1_swedish_ci	None
AlogP	Varchar(500)	No	latin1_swedish_ci	None
AlogP98	Varchar(500)	Yes	latin1_swedish_ci	None
AlogP98_Unknown	Varchar(500)	No	latin1_swedish_ci	None
Formal Charge	Varchar(500)	Yes	latin1_swedish_ci	None

Has Gasteiger Charge	Varchar(500)	No	latin1_swedish_ci	None
Gasteiger Charges	Varchar(500)	Yes	latin1_swedish_ci	None
IsChiral	Varchar(500)	Yes	latin1_swedish_ci	None
LogD	Varchar(500)	No	latin1_swedish_ci	None
Molecular_Formula	Varchar(500)	Yes	latin1_swedish_ci	None
Molecular_Mass	Varchar(500)	No	latin1_swedish_ci	None
Molecular Solubility	Varchar(500)	Yes	latin1_swedish_ci	None
Molecular Weight	Varchar(500)	No	latin1_swedish_ci	None
pKa	Varchar(500)	Yes	latin1_swedish_ci	None
HBA_Count	Varchar(500)	No	latin1_swedish_ci	None
HBD_Count	Varchar(500)	Yes	latin1_swedish_ci	None
Num_AliphaticsDoubleBonds	Varchar(500)	No	latin1_swedish_ci	None
Num_AliphaticSingleBonds	Varchar(500)	Yes	latin1_swedish_ci	None
Num_AromaticBonds	Varchar(500)	No	latin1_swedish_ci	None
Num_AromaticRings	Varchar(500)	Yes	latin1_swedish_ci	None
Num_Atoms	Varchar(500)	No	latin1_swedish_ci	None
Num_Bonds	Int (50)	Yes	latin1_swedish_ci	None
Num_Bonds	Int(100)	Yes	latin1_swedish_ci	None
Num_Chains	Varchar(50)	No	latin1_swedish_ci	None
Num_DopubleBonds	Varchar(500)	Yes	latin1_swedish_ci	None
Num_H_Acceptors_Lipinksi	Varchar(50)	No	latin1_swedish_ci	None
Num_H_Donar_Lipinkshi	Varchar(50)	Yes	latin1_swedish_ci	None
Num_Hydrogens	Int(100)	No	latin1_swedish_ci	None
Num_PiBonds	Varchar(500)	Yes	latin1_swedish_ci	None
Num_Rings	Int(100)	No	latin1_swedish_ci	None
Num_RotatableBonds	Int(100)	Yes	latin1_swedish_ci	None
Molecular_PolarSurfaceArea	Int(100)	No	latin1_swedish_ci	None
Molecular_Surface_Area	Int(100)	Yes	latin1_swedish_ci	None
3D_Desriptor_Warning	Varchar(500)	Yes	latin1_swedish_ci	None
Dipole_Mag	Varchar(500)	No	latin1_swedish_ci	None
AverageBondLength	Varchar(500)	Yes	latin1_swedish_ci	None
Energy	Varchar(500)	No	latin1_swedish_ci	None
Minimized Energy	Varchar(500)	Yes	latin1_swedish_ci	None
RadOfGyration	Varchar(500)	No	latin1_swedish_ci	None
Strain_Energy	Varchar(500)	Yes	latin1_swedish_ci	None
PMI_Mag	Varchar(500)	Yes	latin1_swedish_ci	None
Molecular_3D_PolarSASA	Varchar(500)	No	latin1_swedish_ci	None
Moleular Volume	Varchar(500)	Yes	latin1_swedish_ci	None
OmegaAngle	Varchar(500)	No	latin1_swedish_ci	None
JSME SMILES	Varchar(500)	Yes	latin1_swedish_ci	None
JSME Canonical Smiles	Varchar(500)	No	latin1_swedish_ci	None

Where **PRI:** primary key, generally auto-generated by the database

UNI: this field will take only unique values

NULL: used to represent no value

varchar: MySQL data type, used for storing a string of limited length efficiently

int: MySQL data type, used to represent an integer

Supplementary Table S5: The parameter used for generating 3D conformations of SMMDB ligands.

**Field**	**Type**
Conformation Method	BEST
Maximum Conformations	360
Energy Threshold	20.0
Discard Existing Conformations	False
Separate Conformations	False
Input Forcefield	CHARMm
Maximum Systematic Conformations	1000
Boltzmann Temperature	300
RMSD Cutoff	0.2
Torsion Increment	Default
Sp3-Sp3	120
Sp3-Sp2	120
Sp2-Sp2	180
Conformation Minimization	True
Minimization Algorithm	Smart Minimizer
Minimization Max Steps	2000
Minimization RMS Gradient	0.1
Minimization Forcefield	CHARMm
Minimization Partial Charge Estimation	Momany-Rone
Minimization Dielectric Constant	1
Implicit Solvent Dielectric Constant	80

Supplementary Table S6: List of overlapping small molecule/drug and drug targets in ALS/FTD and FXTAS/FXS disease

Database ID	Drug Name	Status	Disease Name	Pubmed ID	Pubchem Compound ID	Drug Target Name	UNIGENE	Gene Name	NCBI GENE ID	Mode of Action
652	Memantine	Phase II	Amyotrophic Lateral Sclerosis (ALS)	24356417	4054	Glutamate receptor ionotropic, NMDA 3A	654783	GRIN3A	116443	Antagonist
650	Memantine	Clinical Trail Completed	Fragile X-Associated Tremor Ataxia Syndrome(FXTAS)	24345444	4054	Glutamate receptor ionotropic, NMDA 3A	654783	GRIN3A	116443	Antagonist
609	Lithium	Phase II	Amyotrophic Lateral Sclerosis (ALS)	24667005	28486	Glutamate receptor 3	P42263	377070	GRIA3	Potentiator
606	Lithium	Under Investigation	Fragile X syndrome	24814569	28486	Glutamate receptor ionotropic, NMDA 2A	Q12879	411472	GRIN2A	Antagonist
743	NSC311153 (1a)	Under Investigation	Amyotrophic Lateral Sclerosis (ALS)	25132468	432607	r(GGGGCC)exp	493639	C9orf72	203228	Inhibit the protein sequestration to the RNA
742	NSC311153 (1a)	Under Investigation	Fragile X-Associated Tremor Ataxia Syndrome(FXTAS)	22948243	432607	r(CGG)exp	103183	FMR1	2332	Inhibit the protein sequestration to the RNA
